# Remote Monitoring With a Reusable Device Upon Implementation on a Surgical Department (REQUEST Trial)

**DOI:** 10.1002/wjs.70261

**Published:** 2026-02-11

**Authors:** Ephrahim E. Jerry, Arthur R. A. Bouwman, Edith M. G. van Esch, Raoul Richardson, Simon W. Nienhuijs

**Affiliations:** ^1^ Department of Surgery Catharina Hospital Eindhoven Eindhoven the Netherlands; ^2^ Department of Anesthesiology Catharina Hospital Eindhoven Eindhoven the Netherlands; ^3^ Department of Electrical Engineering Signal Processing Systems Eindhoven University of Technology Eindhoven the Netherlands; ^4^ Department of Gynaecology Catharina Hospital Eindhoven Eindhoven the Netherlands; ^5^ Department of Urology Catharina Hospital Eindhoven Eindhoven the Netherlands

**Keywords:** implementation study, nursing workload, postoperative care, surgical ward, vital signs, wearable monitoring

## Abstract

**Background:**

Continuous monitoring of vital signs using wearable devices may improve early detection of postoperative complications and reduce nursing workload. Evidence from real‐world clinical implementation remains limited. This study aimed to answer the question: does the implementation of wearable monitoring in surgical wards reduce nursing workload and is it feasible and acceptable to staff?

**Methods:**

A prospective, single‐center implementation study was conducted on a surgical ward in a large teaching hospital. Nursing workload was assessed using the Integrated Workload Scale (IWS), and usability was evaluated using the system usability scale (SUS). Additionally, staff attitudes were measured with the evidence‐based practice attitude scale (EBPAS). Manual spot checks of vital signs were monitored before and after the implementation of wearable devices (viQtor) for continuous monitoring of heart rate, respiratory rate, and oxygen saturation.

**Results:**

Nursing workload decreased significantly with mean IWS scores dropping from 5.46 ± 1.18 to 3.87 ± 1.38 (*p* < 0.001). A 62.7% reduction in manual spot checks was observed (from 4686 expected to 1748 performed, *p* < 0.001) corresponding to a time saving of 10.1 min per patient per day. The SUS score improved from 74.2 ± 10.1 to 86.0 ± 5.2 (*p* = 0.025). No significant differences were observed in EBPAS scores over time (*p* = 0.43).

**Conclusions:**

Implementation of remote wearable monitoring in surgical wards is feasible, reduces nursing workload, and demonstrates high usability and acceptance among staff. These findings highlight the potential of wearable technology for more efficiency of postoperative care.

**Trial Registration:**

ClinicalTrials.gov: NCT06574867, prospectively registered on 27 August 2024

## 1 Introduction

Monitoring of vital signs is fundamental to the early detection of postoperative complications and remains a cornerstone of patient safety on surgical wards. In current practice, nurses are tasked with performing regular assessments of heart rate, respiratory rate, oxygen saturation, blood pressure, and temperature. Although these observations are indispensable for clinical decision‐making, they are labor‐intensive and represent a significant contributor to nursing workload [1, 2, 3]. Consequently, strategies that alleviate the burden of vital sign monitoring without compromising safety or reliability are of increasing clinical and organizational importance.

Currently, early warning and postoperative monitoring protocols at the hospital ward usually comprise of three manual spot checks of vital signs a day, which are combined in a single aggregated score (Early Warning Score (EWS)) to support timely recognition of clinical decline. However, conventional EWS monitoring provides only intermittent data and imposes substantial workload on nursing staff. Moreover, adherence to these protocols varies considerably between wards and hospitals, further limiting their effectiveness in daily practice [3, 4].

Wearable monitoring devices offer a potential solution by automating vital sign acquisition and reducing reliance on manual spot checks. These devices continuously record parameters such as heart rate, respiratory rate, and oxygen saturation with accuracy comparable to standard bedside monitors and pulse oximeters [2, 5, 6, 7, 8, 9]. By providing high‐frequency, uninterrupted data streams, wearable technology may facilitate earlier recognition of clinical deterioration while supporting more efficient and scalable patient monitoring on surgical wards.

However, beyond their potential clinical utility, wearable devices may also substantially affect workflow and nursing workload. Early implementation studies suggest that wearables can reduce the number of manual assessments and free capacity for other aspects of patient care, although findings have been heterogeneous and overall evidence regarding workflow impact remains limited [10, 11, 12]. Successful adoption depends not only on technical accuracy but also on organizational and behavioral factors, such as staff engagement, training, and integration into daily routines [10, 13].

Wearable monitoring has demonstrated several promising outcomes in clinical research. Prior studies have shown that these devices can reliably capture heart rate, respiratory rate, and oxygen saturation with accuracy comparable to conventional bedside monitors, and in some instances have been associated with earlier recognition of deterioration, fewer unplanned ICU admissions, and reduced length of hospital stay [2, 5, 7, 14, 15, 16].

Despite growing evidence of their accuracy and usability, real‐world implementation of wearable monitoring remains scarce. Most previous investigations have focused on technical validation under controlled conditions rather than clinical integration in routine care [2, 11, 17]. Therefore, this study aimed to evaluate the feasibility of wearable monitoring in a surgical ward setting. Specifically, we conducted a prospective, single‐center implementation study in a large teaching hospital with the primary objective of assessing its impact on nursing workload and secondary objectives focusing on usability and staff acceptance.

## 2 Materials and Methods

### 2.1 Study Design and Setting

A prospective single‐center implementation study was conducted on a surgical ward of a large teaching hospital in the Netherlands. Ethical approval was obtained from the Medical Research Ethics Committee United, and the trial was registered under NCT06574867. The study protocol has been published previously [18]; the present report provides a concise summary of the methodology together with the corresponding results.

### 2.2 Study Population

The study population consisted primarily of healthcare professionals working on the surgical ward. 30 Nurses completed the questionnaires, and three structured focus groups were conducted. For these sessions, participants included nurses (selected based on interest in participation), surgeons, staff from the hospital's information and communication technology department, and one researcher. In parallel, all 622 patients admitted to the ward, irrespective of surgical intervention, were monitored with the wearable device as part of routine care. Standard care on the ward consisted of one Early Warning Score assessment per nursing shift, performed according to the hospital's early warning score (EWS) protocol. For descriptive analyses, one patient day was defined as a calendar day of hospital admission during which the wearable was intended to be in use.

### 2.3 Training and Study Group

Prior to implementation, all nursing staff on the participating surgical ward (general, urology, gynecology; predominantly nononcological patients) received structured instruction provided by the research team and product specialists. Training included hands‐on practice with device application, troubleshooting, and interpretation of monitoring data as well as integration into routine monitoring protocols. Physicians were informed through departmental meetings. All ward nurses and attending physicians were considered part of the study group, as they were directly responsible for applying and interpreting the monitoring system in daily clinical care.

### 2.4 Wearable Device

**FIGURE 1:**
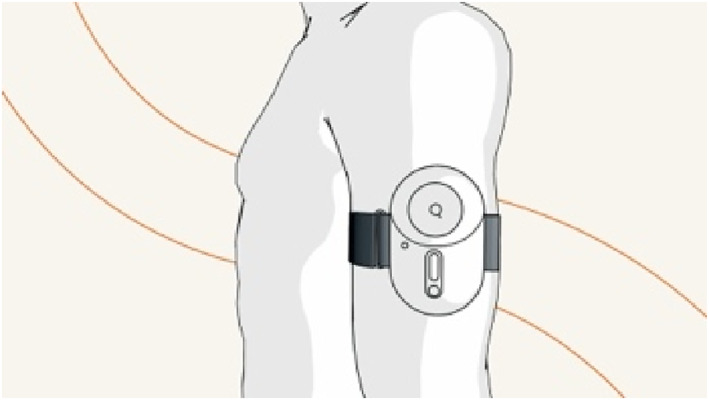
viQtor Device placed on upper arm.

The viQtor (SmartQare, Eindhoven, the Netherlands) is a CE‐marked wearable sensor worn on the upper arm (Figure 1). It continuously measures heart rate, respiratory rate, and oxygen saturation using photoplethysmography. Data were transmitted every 5 minutes to the cloud via a secure mobile network connection without the need for Wi‐Fi or additional hospital infrastructure. During the study period, all patients admitted to the ward were equipped with the wearable regardless of diagnosis or treatment, and the device was intended to be worn throughout their hospital stay.

Earlier studies have shown that the viQtor device provides accurate measurements of vital signs with results comparable to standard clinical monitoring equipment. These findings support its reliability and potential for use in clinical settings, including on surgical wards [19].

### 2.5 Implementation Phases

The implementation was structured in two phases separated by an intermediate evaluation period. This stepwise design allowed for systematic assessment of feasibility, workload impact, usability, and adoption (see Table 1). In Phase 1 (months 1–3), the conventional manual EWS‐based monitoring was continued. In the evaluation month, the device's technical performance and predictive validity were assessed alongside preliminary evaluation of staff experiences. During the second phase (month 5–8), remote wearable monitoring was implemented as the primary modality for vital sign assessment. Manual spot checks were performed only when clinically indicated.

**TABLE 1:** Measurement timetable for psychometric properties.

Objective	Instrument	Month								
		0	1	2	3	4 (evaluation)	5	6	7	8 (evaluation)
Fidelity	SUS						Q		Q	
Acceptance	SC/day				AD				AD	
	SC/patient				AD				AD	
	IWS	Q			Q		Q	Q	Q	
Adoptation	EBPAS	Q			Q				Q	
Appropriate‐ness	SNR								AD	
	ADE								AD	
Feasibility	Thematic analysis				FG			FG	FG	
Time efficiency	Time measurement		M	M	M		M	M	M	
Predictive accuracy	EWS & CREWS		M	M	M	AD & M	AD	AD	AD	AD & M

Abbreviations: AD = administrated data, ADE = Adverse Events, FG = Focus Group, M = manual measurement, Q = Questionnaire, SC/day = Spot Checks per day (the number of spot checks performed each day), SC/patient = Spot Checks per patient (the number of spot checks performed per patient), and SNR = Signal to Noise Ratio.

### 2.6 Integration of Data in Clinical Workflow

During the study, all vital sign data collected by the viQtor wearable device were transmitted to the hospital's electronic health record (EHR) system. Medical staff, including nurses and physicians, were able to review these vital signs directly within the EHR during daily ward rounds. The data were integrated seamlessly into the patient's electronic chart, which allowed for easy access and review without the need for additional logins or systems. The wearable data were displayed alongside other clinical data, such as lab results and previous vital sign readings, making it readily accessible for clinical decision‐making.

Both the CREWS and EWS were used together to inform clinical decision‐making with the wearable data supplementing the manual spot checks performed by nurses when clinically indicated.

### 2.7 Outcome Measures

The primary outcome was nursing workload, assessed with the integrated workload scale (IWS), a validated 9‐point Likert scale.

Secondary outcomes were defined according to Proctor's implementation framework and targeted multiple aspects of feasibility and adoption. Acceptability and adoption were evaluated using the system usability scale (SUS), a 10‐item questionnaire scored on a 0–100 scale that measures perceived usability, and the Evidence‐Based Practice Attitude Scale (EBPAS), a 15‐item tool assessing openness, appeal, requirements, and divergence in relation to evidence‐based interventions. These subjective measures were supplemented with objective data on the frequency of manual spot checks.

Feasibility was assessed by the average time required for vital sign monitoring and by the proportion of successful device use per patient day. Appropriateness was determined by the signal‐to‐noise ratio (SNR) of the collected data and the occurrence of adverse device‐related events. Predictive accuracy was evaluated by comparing continuous remote early warning score (CREWS) values calculated from heart rate, respiratory rate, and oxygen saturation with traditional EWS scores for their ability to identify clinical deterioration. Technical feasibility was assessed through data completeness and integrity metrics, reflecting successful transmission and storage of signals. Finally, implementation fidelity was explored in three structured focus group sessions with nurses and physicians, focusing on experiences, perceived barriers, and facilitators.

### 2.8 Statistical Analysis

Quantitative data were analyzed using IBM SPSS Statistics, version 26.0. Continuous variables were summarized as means with standard deviations (SD) when normally distributed or as medians with interquartile ranges (IQR) when skewed. Categorical variables were reported as frequencies and percentages. To examine differences in outcomes across timepoints, a one‐way ANOVA was conducted for continuous variables with post‐hoc Tukey tests for pairwise comparisons when applicable. Levene's test was used to assess the homogeneity of variances across the groups. For all statistical tests, *p*‐values < 0.05 were considered statistically significant. To examine differences across timepoints, a one‐way ANOVA was conducted for continuous variables, followed by post‐hoc *Games‐Howell* tests to check for significant pairwise differences between timepoints, qualitative data from focus groups were analyzed according to Braun and Clarke's six‐step framework for thematic analysis. Emerging themes included fidelity, feasibility, acceptability, and contextual factors influencing implementation.

## 3 Results

A total of 18 participants took part in the structured focus groups, including nurses, physicians, ICT staff, and a researcher. In addition, 30 nurses completed workload assessments using IWS at multiple time points during the study. During the study period, remote monitoring was applied involving 392 individual patients.

The wearable device was used in all patients, including those with cognitive impairment, unless clinical contraindications, such as significant agitation or discomfort, were present. In cases where patients exhibited signs of agitation (e.g., restlessness or “picking” behavior) or expressed discomfort, nursing staff were permitted to revert to traditional manual spot checks ensuring both the comfort and safety of the patient.

### 3.1 Primary Endpoint

**FIGURE 2:**
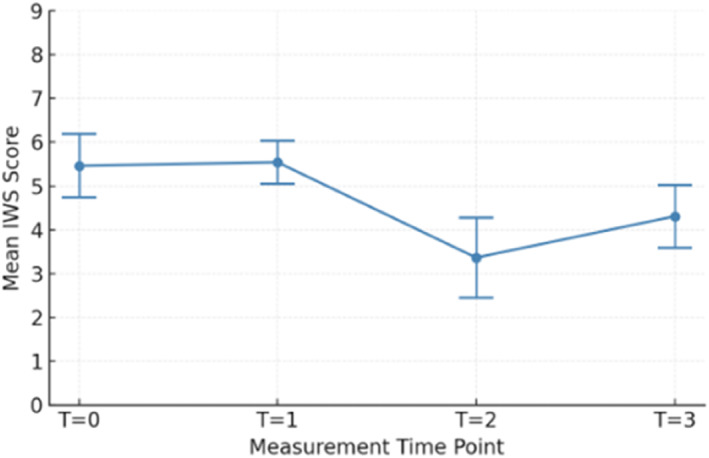
Nursing workload over time. *T* = 0 (baseline), *T* = 1 (early implementation), *T* = 2 (mid implementation), and *T* = 3 (end of implementation). Values are shown as mean scores with 95% confidence intervals.

Across four time points, a one‐way ANOVA revealed a significant effect of time on nursing workload, F (3, 86) = 7.30, *p* < 0.001, and *η* ^2^ = 0.20 (Figure 2). Post‐hoc Tukey tests showed that workload was significantly lower at T2 compared with T0 (*p* = 0.001), T1 (*p* = 0.001), and T3 (*p* = 0.001), whereas no significant differences were found among T0, T1, and T3 (all *p* > 0.19).

Mean IWS scores differed significantly between timepoints with the lowest mean observed at T2 (3.87 ± 1.38) and higher means at T0 (5.46 ± 1.18), T1 (5.61 ± 1.38), and T3 (4.62 ± 1.45).

### 3.2 Secondary Endpoints

The SUS increased from a mean score of 74.2 (SD 10.1) at the first focus group session to 86.0 (SD 5.2), at all timepoints, the SUS score was above 70 corresponding to the threshold for excellent usability. The one‐way ANOVA analysis showed a significant overall effect of time on usability scores, *F* (2, 16) = 4.71, *p* = 0.025, *η* ^2^ = 0.37. There were no significant differences between the timepoints for the SUS scores (all *p* > 0.30).

Across 1562 monitored patient days, the average number of manual spot checks decreased by 1.88 per patient per day after the introduction of remote monitoring. This corresponds to a 62.7% reduction in the total number of EWS measurements, from an expected 4686 to 1748 performed, a chi‐square test confirmed that this reduction was statistically significant with a chi‐square value of 1842.05 (*p* < 0.001). This reduction translated to a saving of approximately 10.1 min per patient per day equivalent to a total of 263 nursing hours saved during the 9‐month study period.

The mean EBPAS total score was 2.99 (SD 0.55) at *T* = 0, 3.07 (SD 0.43) at *T* = 1, and 2.83 (SD 0.32) at *T* = 2 (Table 2).

**TABLE 2:** Evidence‐based practice attitude scale outcomes across implementation phases.

Domain	*T* = 0	*T* = 1	*T* = 2
Appeal	3.21 ± 0.55	3.31 ± 0.42	3.12 ± 0.36
Requirements	3.33 ± 1.17	3.08 ± 0.88	2.63 ± 0.62
Openness	2.46 ± 0.76	2.58 ± 0.36	2.35 ± 0.39
Divergence	2.07 ± 0.43	2.40 ± 0.59	2.10 ± 0.49
Total score	2.99 ± 0.55	3.07 ± 0.43	2.83 ± 0.32

*Note:* Values represent mean ± SD. *T* = 0 indicates baseline (start of the study), *T* = 1 the midpoint during implementation, and *T* = 2 the end of the study period.

The sub scores for Requirements, Openness, and Divergence showed similar trends with no significant differences between timepoints. A one‐way ANOVA was conducted to examine differences in EBPAS scores across the three timepoints (*T* = 0, *T* = 1, *T* = 2). Levene's test confirmed homogeneity of variances for all scores (*p* > 0.05). For the total EBPAS score, no significant differences were observed between timepoints, F (2, 27) = 0.88, *p* = 0.43. Similarly, no significant differences were found for any of the subscores (Requirements, Openness, and Divergence) with *p*‐values of 0.26, 0.55, and 0.28, respectively.

### 3.3 Alarm Frequency and Predictive Performance

The introduction of the viQtor wearable system led to a higher alarm frequency (R3EWS: 7.8%) compared to the manual MEWS system (5.9%) reflecting an increased sensitivity for detecting clinical deterioration. This increase in alarm frequency was associated with a higher sensitivity (0.172 vs. 0.120) but did not result in an increased number of ICU admissions or unnecessary interventions, suggesting that the system did not contribute to overtriage.

### 3.4 Thematic Insights From Focus Group Interviews

In addition to the quantitative data, three focus group discussions were held to gain a deeper understanding of staff experiences and perspectives on the implementation of wearable monitoring. These discussions aimed to capture the nuances of usability, workflow integration, and staff attitudes, which are crucial for the successful adoption of such technology in clinical practice. The focus groups provided valuable qualitative data that complement the quantitative findings, particularly regarding staff acceptance, responsibility, and the integration of the wearable system into daily routines. Table 3 presents a thematic analysis of the feedback gathered, which highlights key factors influencing the adoption and sustained use of the wearable device by the nursing staff.

**TABLE 3:** Thematic analysis of focus group data.

Theme	Focus group 1 (after 4 months)	Focus group 2 (after 6 months)	Focus group 3 (after 8 months)
Theme 1: Acceptance and user experience	Most nurses indicated a positive attitude toward using viQtor. They found the system user‐friendly and especially valued the time savings and convenience during morning and afternoon rounds. Decisions to use the system were mainly based on practical considerations per patient.	Nurses reported that acceptance and daily use of viQtor had greatly improved. Almost all patients were now connected by default. Time savings, particularly during morning rounds, were seen as a major advantage.	Nurses described viQtor as intuitive and expressed growing trust in the recorded vital signs. They perceived less need for additional manual measurements as long as the device seemed reliable.
Theme 2: Responsibility and ownership	Nurses expressed a strong sense of responsibility for following up on viQtor values. However, doubts remained about how consistently this was done, especially among colleagues less engaged in the implementation.	Nurses felt responsible for correct use of viQtor, such as connecting, disconnecting, and checking battery status. Some colleagues, however, sometimes forgot to use or disconnect the system, which caused frustration. Participants also showed initiative in defending the use of viQtor toward physicians and colleagues, although this required effort.	Nurses expressed frustration about missing data, long delays in confirming device connection, and limited access to guardian. A specific period with multiple technical malfunctions led to reluctance in use.
Theme 3: Protocol adherence and routines	There was uncertainty about when and how often viQtor values should be checked. Some suggested including this in the nursing plan, for example as a fixed activity during morning rounds. Motivation seemed partly dependent on individual effort and involvement in the project.	Since viQtor was added to the nursing activity plan (to be checked three times a day), protocol adherence improved. Measurements were generally reviewed in the morning, afternoon, and evening, although afternoon checks were still less consistent.	Although most nurses routinely reviewed viQtor data, some colleagues occasionally forgot. A more clear standard seemed desirable.
Theme 4: Contraindications and technical limitations	Certain patient groups, such as people with dementia or very thin upper arms, were sometimes excluded from viQtor use. Doubts were also raised about data reliability under specific clinical conditions.	Nurses used viQtor almost always. Only in cases of severe restlessness, picking behavior, or a suspected allergic reaction was use limited. There was some doubt regarding an incident with a possible skin reaction.	Users noted that incorrect application (too loose, reversed) was a major cause of data loss. There were doubts about whether everyone attached the sensor correctly, especially after showering or reconnecting. The size of the device was repeatedly mentioned.
Theme 5: Feedback systems and monitoring needs	Nurses observed that the system provided few triggers to actively review measurements. Suggestions included automatic alerts for abnormal values or integration into the electronic hospital registration.	Guardian was rarely used by nurses. They felt that their electronic hospital registration was sufficient and that real‐time monitoring or trend graphs were usually unnecessary, though some saw added value in specific cases.	There was a strong demand for faster data visibility after connecting the device (sooner than 4 hours). Suggestions included a central ward screen or a visual dashboard showing battery status and data transmission.
Theme 6: Sustainability and future perspective	Nurses saw potential for continued use of viQtor, provided the whole team remained involved and clear guidelines were available. Acceptance increased as they began to experience the benefits	Nurses were unanimously enthusiastic about continued use of viQtor, provided sufficient budget and departmental support were ensured. They particularly appreciated the time efficiency and patient‐centered approach.	Participants expressed that expansion to seven beds on the ward would be desirable. The system was seen as particularly time‐saving during busy morning shifts.

Nurses reported an increase in acceptance of the viQtor system over time with initial appreciation for its ease of use and time‐saving benefits. Over time, use became more routine with growing trust in the recorded vital signs. Acceptance and user experience improved though frustrations arose with inconsistent use by some colleagues, highlighting the responsibility and ownership theme. Integration into the nursing activity plan improved adherence to reviewing the data from the wearable device but adherence to consistently reviewing the data remained less reliable in the afternoons, highlighting the need for clearer routines for data review during this time.

Contraindications and technical limitations were noted, such as exclusion of patients with dementia and issues with device placement and battery status. Nurses expressed a desire for faster more visual feedback, reflecting feedback systems and monitoring needs. Overall, they were enthusiastic about continued use of the device, provided there was sufficient support and equipment, supporting the sustainability and future perspective theme.

## 4 Discussion

This study evaluated the implementation of viQtor, a wearable device for remote monitoring of vital signs on a surgical ward. The principal findings were that implementation was feasible, nursing workload decreased over the study period, and usability reached a high and excellent level. Qualitative analyses further demonstrated increasing acceptance, integration into daily routines, and the importance of clearly defined responsibilities. Notably, professional attitudes toward evidence‐based practice remained stable, indicating that short‐term implementation does not necessarily influence underlying professional values. Although continuous vital sign monitoring has been widely investigated and has demonstrated potential advantages over intermittent EWS‐based monitoring, the present results show that implementation is feasible and supports healthcare professionals in their clinical work by reducing workload.

Our findings are consistent with previous implementation studies with several key contributions and unique aspects. Firstly, the viQtor, a reusable wearable device, was found to be feasible and well‐integrated into the clinical workflow, providing continuous monitoring of vital signs, including heart rate, respiratory rate, and oxygen saturation. These features allow for a more comprehensive assessment of patient status compared to previous disposable devices such as Healthdot. Our study demonstrates that the viQtor's ability to integrate seamlessly with the electronic health record enhanced workflow and minimized manual data entry. This is consistent with findings by Leenen et al., who highlighted that workflow integration is crucial for the adoption of wearable technologies in clinical settings [10].

The impact on nursing workload was particularly notable with a 62.7% reduction in manual spot checks and a time saving of 10.1 min per patient per day. These results align with earlier studies that have shown similar reductions in workload following the implementation of wearable devices for continuous monitoring (Patel et al.). By reducing manual spot checks, nurses were able to allocate more time to direct patient care, which is an important outcome in busy surgical wards [7, 13, 20].

Usability was also a key focus of our study with SUS scores increasing from 74.2 to 86.0 over the study period, indicating a significant improvement in device acceptance. This improvement is in line with the findings of Reijmers et al., who reported high usability scores for wearable devices when integrated into clinical settings. Nurses in our study expressed growing trust in the viQtor highlighting its reliability and ease of use over time. However, some challenges regarding protocol adherence remained particularly in the afternoon shifts, as nurses expressed frustration with inconsistent use by colleagues. This issue of ownership and responsibility aligns with Leenen et al., who noted the importance of engagement and clear guidelines to ensure consistent use of wearable technologies [13, 21].

Key strengths of this study include its mixed‐methods design integrating quantitative measures (IWS, SUS, EBPAS) with qualitative insights from focus groups as well as the repeated assessments that enabled evaluation of evolving staff attitudes over time. Importantly, implementation was deliberately designed to remain as close as possible to the existing workflow, focusing on reducing nursing workload rather than altering established care processes.

Also one of the key findings of our study is that the implementation of the viQtor wearable device led to a significant reduction in nursing workload primarily by automating the monitoring of vital signs. However, it is important to emphasize that this reduction in workload did not result in the withdrawal of essential monitoring. Rather than replacing clinical assessments, the device augmented the existing workflow by providing continuous data, which were reviewed alongside traditional early warning scores (EWS). This ensured that the healthcare team was still able to make timely and informed decisions, while reducing the repetitive burden of manual vital sign checks. By maintaining a dual system of automated and manual monitoring, we were able to enhance the efficiency of care delivery without compromising patient safety or clinical oversight.

Several limitations should be acknowledged in this study, which may influence the generalizability of the findings and the interpretation of the results. Firstly, this was a single‐center study with a relatively small sample size especially for survey‐based outcomes. Although a total of 622 patients were monitored during the study period, the sample size remains modest for drawing broad conclusions particularly with respect to questionnaire‐based measures (e.g., IWS, SUS, EBPAS). Previous studies have shown that smaller sample sizes can limit the power to detect subtle effects, particularly when evaluating subjective experiences such as user acceptability and staff attitudes [13, 22].

Secondly, although the initial findings suggest significant reductions in workload and high usability, a longer follow‐up period would allow us to assess whether these effects persist over time. Furthermore, a longer follow‐up would provide a clearer picture of the sustainability of the observed workload reductions and usability gains, as short‐term benefits might not always translate into long‐term clinical practice changes.

Another limitation identified throughout the study were the technical challenges encountered during the implementation phase, which disrupted workflow and occasionally undermined staff trust in the system. Specifically, there were multiple instances where vital sign measurements failed to be transmitted to the Electronic Health Record (EHR) due to server‐side disruptions. These disruptions were often resolved within a day, but they caused temporary data gaps, resulting in missed readings and affecting the reliability of the system in real‐time clinical use. It is essential to address these issues through technical optimization of the system, including redundancy in data transmission and storage processes, to ensure continuous data availability and prevent workflow interruptions. In addition, the battery life of the viQtor device was a recurrent issue, when devices were left unmonitored. Developing a user‐friendly interface for checking battery status and ensuring adequate device placement before shifts will help improve the reliability of the technology in everyday clinical use. Finally, the EBPAS instrument may not have been sufficiently sensitive to detect localized changes in workflow‐related attitudes, a limitation that has been described previously in the implementation science literature [23].

Future research should focus on further optimizing workflow integration. In our study, vital signs were already automatically transferred into the electronic health record, which facilitated adoption. The next step is to combine such integration with clinical decision support systems to further improve usability, efficiency, and clinical responsiveness. Larger multicenter trials are required to confirm the effects on patient safety and clinical outcomes [17]. Importantly, cost‐effectiveness analyses and exploration of predictive analytics using artificial intelligence [15, 24] may further demonstrate the value of wearable monitoring in hospital care. Longer follow‐up periods will be necessary to assess sustainability and to explore how professional attitudes evolve with prolonged exposure.

This study demonstrates that deploying wearable monitoring devices with reusable components on surgical wards is feasible, leading to decreased nursing workload and favorable usability evaluations. Although professional attitudes largely remained unchanged, qualitative data indicated increasing acceptance and enthusiasm among nursing staff. Overall, these results suggest that wearable monitoring can effectively support clinical practice when technical reliability, integration with existing workflows, and sustainable institutional support are ensured. Further multicenter investigations with extended follow‐up periods are recommended to validate these outcomes and inform wider implementation of wearable monitoring in surgical care.

## Author Contributions

**Ephrahim E. Jerry:** conceptualization, methodology, investigation, data curation, formal analysis, writing – original draft, project administration. **Arthur R. A. Bouwman:** conceptualization, methodology, formal analysis, supervision, writing – review and editing. **Edith M. G. van Esch:** investigation, resources, writing – review and editing. **Raoul Richardson:** investigation, resources, writing – review and editing. **Simon W. Nienhuijs:** conceptualization, supervision, writing – review and editing.

## Funding

The authors have nothing to report.

## Conflicts of Interest

Study wearables were provided by SmartQare (Eindhoven, the Netherlands).

## Data Availability

The data that support the findings of this study are available from the corresponding author upon reasonable request.
